# Adsorption Heat Storage: State-of-the-Art and Future Perspectives

**DOI:** 10.3390/nano8070522

**Published:** 2018-07-12

**Authors:** Salvatore Vasta, Vincenza Brancato, Davide La Rosa, Valeria Palomba, Giovanni Restuccia, Alessio Sapienza, Andrea Frazzica

**Affiliations:** CNR—ITAE, Istituto di Tecnologie Avanzate per l’Energia “Nicola Giordano”, Via Salita S. Lucia sopra Contesse 5, 98126 Messina, Italy; vincenza.brancato@itae.cnr.it (V.B.); davide.larosa@itae.cnr.it (D.L.R.); valeria.palomba@itae.cnr.it (V.P.); giovanni.restuccia@itae.cnr.it (G.R.); alessio.sapienza@itae.cnr.it (A.S.); andrea.frazzica@itae.cnr.it (A.F.)

**Keywords:** adsorption, heat storage, thermo-chemical, zeolite, silica gel, adsorbent materials

## Abstract

Thermal energy storage (TES) is a key technology to enhance the efficiency of energy systems as well as to increase the share of renewable energies. In this context, the present paper reports a literature review of the recent advancement in the field of adsorption TES systems. After an initial introduction concerning different heat storage technologies, the working principle of the adsorption TES is explained and compared to other technologies. Subsequently, promising features and critical issues at a material, component and system level are deeply analyzed and the ongoing activities to make this technology ready for marketing are introduced.

## 1. Introduction

**Figure 1:**
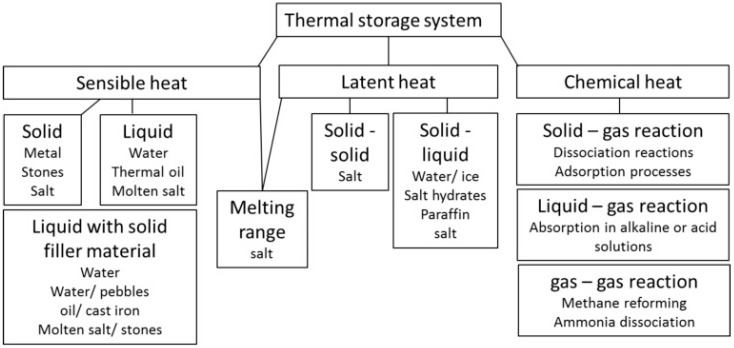
Thermal energy storage (TES) technologies [5].

One of the key technologies for boosting the diffusion of renewable energies and for developing efficient energy systems is Thermal Energy Storage (TES). Indeed, the employment of TES has the potential to overcome the existing mismatch between energy production and demand for discontinuous energy sources (e.g., solar thermal) and/or variable loads (e.g., thermal energy demand in buildings). It is therefore evident that this component is gaining a crucial role in the development of highly efficient thermal energy systems [1]. The three main technologies for thermal energy storage are: sensible, latent and thermo-chemical heat storage, as shown in Figure 1.

In a sensible heat storage, thermal energy is stored as a function of the temperature difference only. The amount of stored energy depends both on the specific heat and on the temperature difference between the charge and discharge phase. The heat storage media can be either liquid or solid. The most common example of sensible heat storage media is water. Indeed, it represents the typical medium employed for heat storage at temperatures below 100 °C, as it couples its abundance, cost-effectiveness, and non-polluting features with good thermodynamic characteristics, such as high specific heat and good heat transfer efficiency within the operating ranges. However, its working temperature range is limited between 0 and 100 °C, and it presents corrosive behavior.

The main advantages of sensible heat storages are related to the simple design, cost-effectiveness, and wide temperature range of applications. Nevertheless, they are characterized by low heat storage density and degradation of the stored energy due to heat dissipation through the environment. This means that they cannot be efficiently used for storing heat for medium/long-term periods [2].

In the latent heat technology storage, thermal energy is mainly stored by exploiting the latent heat of the phase transition of the heat storage medium. The phase change process can involve either a solid/liquid transition (i.e., melting/solidification process) or a solid/solid transition (i.e., transition from one crystal structure to a different one). The most common class of materials employed as latent heat storage media are: salts, water, hydrated salts and paraffins, which usually undergo a solid/liquid transition. The advantages of such a technology are higher heat storage density compared to sensible systems, the possibility to store energy in a narrow temperature range, and the possibility to employ the phase transition to smooth temperature fluctuations (e.g., in building envelope applications). The main disadvantages are related to the slow kinetics of the phase transition, which limits the charging/discharging power, the instability of materials undergoing several melting/solidification processes, and the presence of a volume variation when passing from the solid to liquid phase, which needs to be carefully considered during the design process. Furthermore, latent heat storage suffers from heat dissipation to the environment, which limits its application for long-term heat storage [3].

The thermo-chemical technology is based on the reversible reaction occurring between two components and is associated with a high amount of energy. These reactions can be either chemical or physical. The main limits are related to the very slow reaction kinetics, due to the high energy associated with the process, as well as heat and mass transfer diffusion resistance within the material. Physical reactions are typical of sorption applications, where a refrigerant (e.g., water, ammonia) reacts with a sorbent, which can be either liquid (absorption systems), or solid (adsorption systems). As this technology is based on physical reaction, it generally needs lower charging temperatures (i.e., 70–150 °C) and is characterized by lower reaction enthalpies compared to the chemical reactions. Accordingly, they are characterized by faster kinetics but lower heat storage densities [4].

The present paper will focus on the analysis of the state-of-the-art and future perspectives of the adsorption heat storage systems, mainly for domestic and tertiary sectors.

## 2. Principles of Adsorption Heat Storage

**Figure 2:**
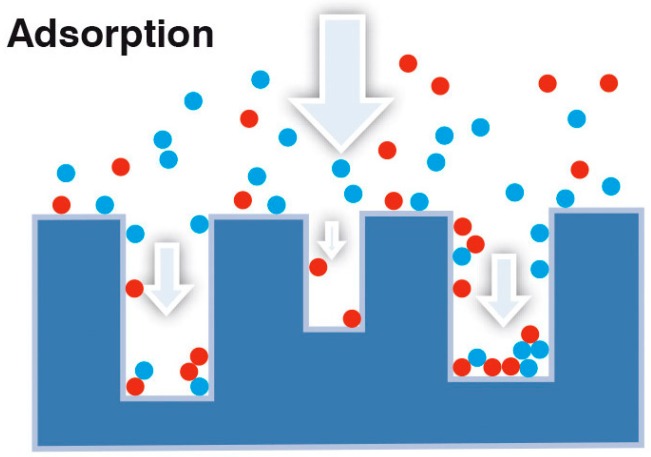
Adsorption of refrigerant over the external surface of an adsorbent solid material.

Adsorption heat storage belongs to the wider class of thermo-chemical heat storage. This technology is based on the interaction between a liquid sorbate, usually water, and a solid sorbent (e.g., zeolites, silica gels, activated carbons). This interaction occurs between the sorbate molecules and the available surface of the solid, as represented in Figure 2.

**Figure 3:**
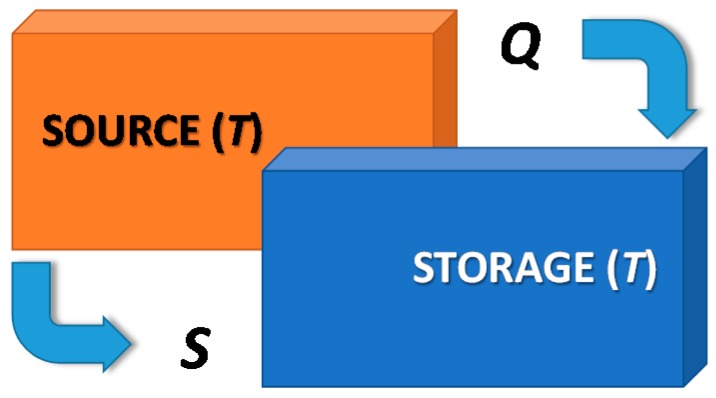
Direct heat storage, charging phase.

In order to explain the working principle of an adsorption heat storage, it is necessary to distinguish between direct and indirect heat storage methodologies [6]. The direct heat storage, as represented in Figure 3, is the typical technology employed to store heat in both sensible and latent form.

In this case, there is a coupled heat and entropy flux from the heat source to the storage, at temperature T. Accordingly, since heat and entropy are closely related, Q = T∙S, this means that the heat storage capacity depends on the temperature level and on the entropy content. Due to the temperature difference between TES (either hot or cold storage) and ambient temperature, there is a continuous loss of heat-storage capacity over time, due to the reduction of ΔT between stored heat and ambient temperature. Accordingly, it is possible that below certain levels of ΔT, the stored heat is no longer useful for practical applications (e.g., domestic hot water, space heating).

**Figure 4:**
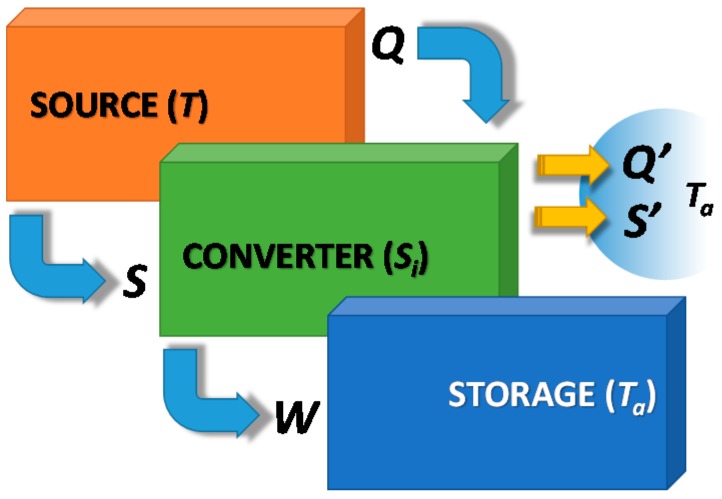
Indirect heat storage, charging phase.

In contrast, an indirect TES, as represented in Figure 4 for the charging process, can overcome this limitation, converting heat into a different form of energy (e.g., mechanical, chemical), which is stored without any limitation (i.e., no heat losses to the ambient). As this technology is based on an energy conversion process, the converter needs to be connected to an external sink (e.g., the ambient) through which the produced waste heat and entropy, due to irreversibility, must be dissipated. The reverse phenomena, which exploits heat and entropy flux from the ambient to convert the stored energy into useful heat again, represents the discharging phase. It is therefore evident that this technology needs to be connected to two different sources/sinks, which makes the TES an indirect process, like a heat pump.

Adsorption heat storages belong to the indirect TES class. Indeed, in this case, heat is employed to drive a desorption process, which means that energy is stored in the form of adsorption potential energy. In this way, heat is stored and conserved until the refrigerant fluid (adsorbate) is kept separate from the adsorbent.

Generally, there are two system configurations for adsorption TES: closed- and open-cycle.

**Figure 5:**
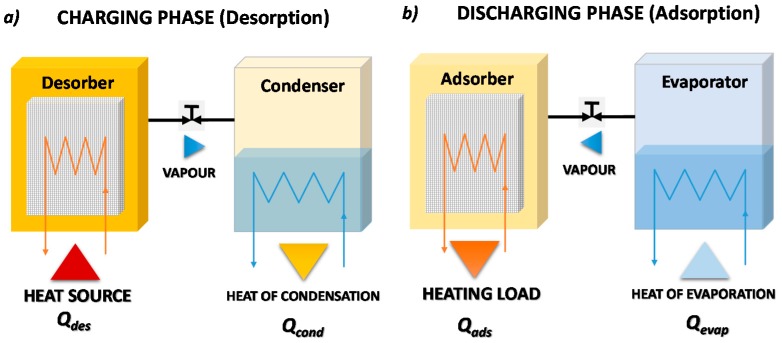
A closed adsorption heat storage cycle: (**a**) charging phase; (**b**) discharging phase.

Figure 5 reports the working phases of a closed adsorption TES. During the charging phase as shown in Figure 5a, the adsorber, in which the adsorbent material is saturated with adsorbate, is regenerated exploiting heat, Q_des_, coming from the heat source. The desorbed vapor condenses in the condenser, and the heat of condensation, Q_cond_, is dissipated into the ambient or delivered to the load, if the temperature level is adequate. Once the charging process is completed and the adsorbent material is dry, the connection between condenser and adsorber is closed. In this condition, the system can conserve the stored energy for an indefinite time, since the thermal energy is stored as the adsorption potential between adsorbate and adsorbent material. To recover the stored thermal energy, as shown in Figure 5b, the connection between the liquid adsorbate reservoir, which in this phase acts as an evaporator, and adsorber is opened once again. During this discharging phase, the adsorbate is evaporated, adsorbing heat from the ambient, Q_evap_, before the vapor flows into the adsorber, where it is adsorbed. Since the adsorption process is exothermic, the heat of adsorption, Q_ads_, is released to the load.

Clearly, this process is defined as closed since the adsorbate is continuously condensed/evaporated in a closed system without any mass exchange with the ambient.

**Figure 6:**
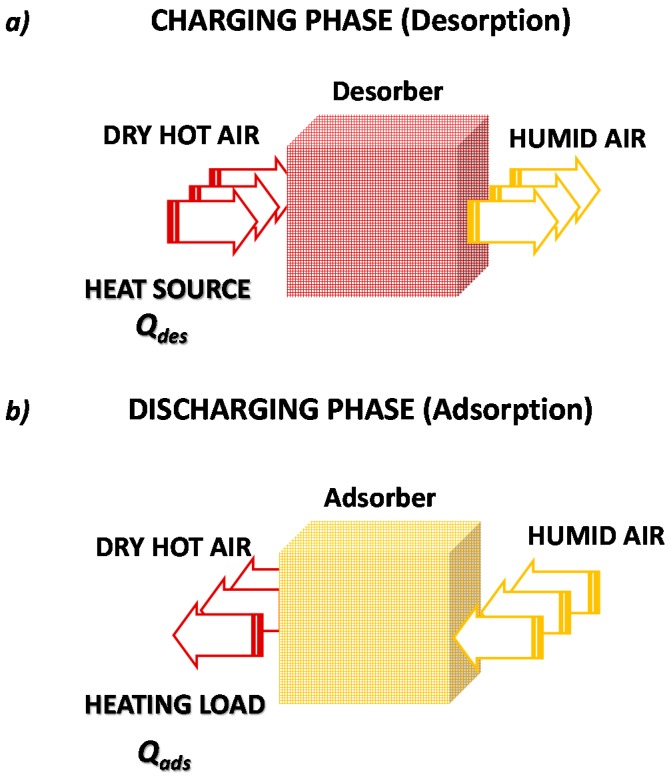
An open adsorption heat storage cycle: (**a**) charging phase; (**b**) discharging phase.

Contrastingly, the open adsorption TES system, represented in Figure 6, continuously exchanges mass (adsorbate) with the ambient. The two charging/discharging phases are, in fact, similar to those already described for the closed cycle. In this case, heat is provided and extracted by fluxing air through the adsorbent bed. Specifically, during the charging/desorption phase, a hot and dry air flux enters the storage, causing the desorption of adsorbed water, and exits at a lower temperature and higher humidity content.

During the discharging/adsorption phase, a humid and cooled air flux is provided to the dry adsorbent, which triggers the adsorption and consequent release of the stored heat. The stored heat is released as hot and dry air flux exiting the system. Some clear differences must be highlighted between closed and open adsorption TES.

- Open adsorption TES can employ only water as adsorbate, since they exploit the moisture of the ambient air as working fluid. On the contrary, closed adsorption TES can use a different adsorbate, even if the most widely employed is water, owing to its high latent heat as well as its environmental friendly feature.
- Open adsorption TES strongly depends on the external ambient conditions. This means that the higher the moisture content of the external air, the higher the heat storage density that can be achieved.
- Closed adsorption TES, if employed for short-term heat storage, can also exploit the heat-pumping effect, related to the energy recovered from the condensation of water vapor during the charging phase. In this manner, the energy storage density is enhanced. On the contrary, for the open adsorption TES, the heat of condensation is dumped to the ambient and not recovered.
- Closed adsorption TES are usually more complex systems, since they employ different heat exchangers to provide/extract heat to the adsorber and the evaporator/condenser. Furthermore, working in a closed cycle, they need to keep a saturated adsorbate atmosphere, meaning that any air leakage must be prevented, making systems more complex and expensive. On the contrary, open adsorption TES are less complex and expensive systems, which seem more suitable for long-term heat storage.

## 3. State-of-the-Art

Adsorption TES is considered to be quite a promising technology both for seasonal and daily storage applications, nevertheless, its commercial diffusion is still not fully developed, mainly due to its cost and the lack of technical knowledge at a system level. This means that there is still need for development and research, in order to make the technology commercially competitive. The research activities in the field can be divided into three levels: Materials, components, and systems.

### 3.1. Adsorbent Materials

Development of adsorbent materials for adsorption TES is strongly related to the adsorbate to be employed. As the most common adsorbates are water and ammonia, the following sections summarize the research activities on adsorbents developed for these kinds of adsorbates.

#### 3.1.1. Silica Gels

Silica gels historically represent one of the most employed adsorbent materials for water vapor adsorption. In fact, they represent a less expensive option for adsorption TES applications and can be easily employed for heat sources at temperatures lower than 100 °C (e.g., flat-plate solar thermal collectors). It is important to highlight that the porous structure of silica gels for closed adsorption TES must be completely different from that which is employed for open adsorption TES. Indeed, as in a closed system the adsorption/desorption process usually occurs in a limited partial pressure range (e.g., between 0.1 and 0.3 p/p_0_), it is necessary to have silica gels with highly microporous structures, capable of exchanging high quantities of water vapor. On the contrary, in an open adsorption TES, since the working partial pressures are usually higher, a mesoporous silica gel can be also employed, due to the capillary condensation phenomena that occurs within this working range.

As will be described later in this work, despite their competitive cost and wide availability, silica gels showed too low a heat storage capacity, which often leads to an experimental heat storage density even lower than that of water [7,8]. Nevertheless, they still represent a possible option if employed for long-term heat storage applications. With regard to such applications, the cost can become the main selection criteria. Furthermore, their application as a host matrix for composites is considered an interesting alternative.

#### 3.1.2. Classical Zeolites

Zeolites are crystalline alumino-silicates, characterized by a high specific surface area (i.e., about 800 m^2^/g) and wide microporous volumes, which make these materials perfectly suitable for water vapor adsorption. Owing to their porous structure, zeolites are usually highly hydrophilic, which allows them to obtain high adsorption capacities even at low partial pressures. This high affinity with water, of course, is reflective of strong bonding that requires higher temperatures to be broken compared to silica gels (i.e., more than 150 °C). Zeolites type A, 13X and Y are the most common classical synthetic zeolites employed for adsorption heat storage. These materials are mostly used for open adsorption TES, since, in order to get enough energy storage density, they must be regenerated at high temperatures, making air the most effective heat transfer medium. Thus, due to the required high operation temperature, they are usually employed for industrial waste heat recovery and storage [9].

Owing to their crystalline structure, as opposed to the amorphous structure of silica gels, they can guarantee a higher long-term hydrothermal stability; offering a more reliable option for applications where several adsorption and desorption cycles are expected.

#### 3.1.3. Zeo-Like Materials

More recently, several new microporous adsorbents have been proposed for TES applications. They are often referred to as zeo-like materials, as their crystalline structure is somewhat similar to those of classical zeolites. The two classes that showed the most promising features are the aluminophosphates (AlPOs) and the silico-aluminophosphates (SAPOs). Indeed, in contrast to other classical adsorbents, these materials show a partially hydrophobic behavior, that is reflected in an S-shaped adsorption isotherm. This is an advantageous characteristic, that allows a high amount of water vapor exchange to be obtained in a narrow range of partial pressure. Accordingly, since the overall heat storage capacity is highly dependent on the water vapor exchange, these materials can guarantee very high heat storage capacities.

Among these two classes, the most attractive materials are known as AlPO-18 and SAPO-34, as reported by several authors [10]. Particularly, the research on these materials lead to the first commercial adsorbent specifically developed for closed adsorption systems (e.g., for heating, cooling and storage applications). It is known as AQSOA Z02, and is produced and commercialized by Mitsubishi Plastic Inc. (Chiyoda-ku, Tokyo) [11].

#### 3.1.4. Metal Organic Frameworks

Metal Organic-Frameworks (MOFs) represent a new, emerging class of adsorbent materials [12]. These materials, still in an early stage of development, are considered the future of adsorption TES. Due to their structure, made up of metal ions interconnected by organic macro-molecules, it is possible to select several different compositions; giving infinite possibilities to obtain the ideal adsorbent material by simply adjusting the synthesis procedure. Indeed, they are usually characterized by a high specific surface area (i.e., higher than 2000 m^2^/g), which guarantees the ability to reach higher adsorption capacities compared to other adsorbent classes. Higher adsorption capacities are also achieved by tuning of the pore sizes, according to the adsorbate and the working range. Nevertheless, as previously mentioned, this class is still far from practical application, due to two main reasons: Their high cost, related both to the small amount currently produced and to the cost of raw materials, and their hydrothermal stability, that still requires thorough investigation.

#### 3.1.5. Activated Carbons

Activated carbons are carbonaceous adsorbent materials, obtained from different possible precursors (e.g., coconut shells, wood, coal), characterized by a wide specific surface area (1200–1300 m^2^/g) and microporous volume. These materials are typically employed as adsorbents of ammonia and alcohols for adsorption TES, owing to the high affinity demonstrated towards these adsorbates. Owing to the competitive cost and wide commercial availability, they are considered as a promising option for TES applications. However, they can be employed only for closed adsorption TES, since their affinity towards water vapor is quite limited. Furthermore, there are no examples in the literature of the use of these materials in TES to date. As for the case of silica gels, they have been mainly investigated as possible substrates and matrices for composite adsorbents, primarily exploited to increase the poor thermal conductivity of adsorption materials.

#### 3.1.6. Composite Sorbents

**Figure 7:**
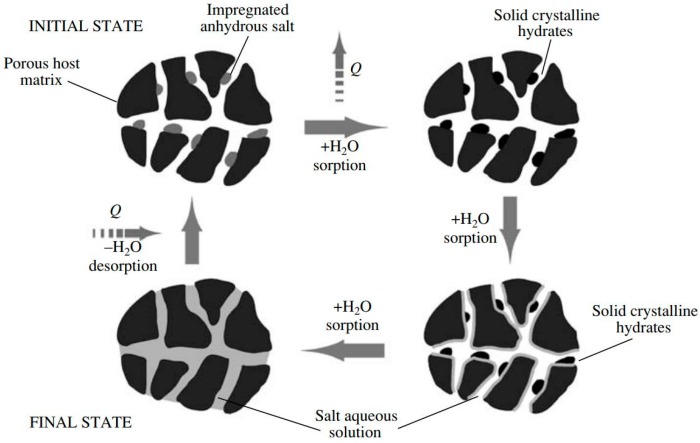
The working principle of a composite adsorbent [13], with permission from Springer Nature, 2007.

Composite sorbents represent a hybrid method to enhance the sorption ability of materials under the typical working boundary conditions of adsorption TES [13]. Indeed, they are based on the embedding of inorganic salt (e.g., CaCl_2_, LiCl, LiBr) inside a host porous structure (e.g., silica gel, vermiculite). This concept was invented by the Boreskov Institute of Catalysis, in an attempt to exploit the absorption ability of certain kinds of salts, while avoiding one of their main limitations, that is, the excessive mass transfer limitation induced by the agglomeration of the salt when it is employed in bulk. Indeed, as depicted by the working principle reported in Figure 7, the reaction between salt and adsorbate is always confined inside the pores of the host matrix, this makes the adsorption/desorption more stable and less affected by the adsorbate diffusion phenomena. Furthermore, the proper selection of the salt and the pore size of the host matrix offers the ability to nano-tailor the achievable adsorption properties of the synthesized material. Owing to the wide availability of inorganic salts, capable of reacting with different adsorbates, it is possible to synthesize composite sorbents that can be used with a large number of adsorbates. Typical examples are the well-known Selective Water Sorbents, SWSs, which represent the wider class of composite materials, specifically developed for water adsorption.

The literature is full of several different developed composite sorbents for TES applications. They often demonstrate an attractive performance, in terms of heat storage density, especially on a thermodynamic basis. The main issues displayed by these materials are related to possible salt leakage from pores and slow kinetic behavior, due to the chemical reaction occurring between salt and adsorbate.

### 3.2. Adsorption Material Heat Storage Calculations

An interesting comparison among the several adsorbent materials introduced above is reported in [14]. The calculations are only performed for closed systems with water as the adsorbate, as these are still the most widely employed adsorption TES under investigation. In the present paragraph, some of the main obtained outcomes are summarized. Generally, in order to calculate the most effective adsorbent material for TES, the main parameter to be investigated is represented by the integral enthalpy of adsorption, which can be easily calculated, as reported by the following equation:

(1) $$H_{ads}=∆H_{ads}\;\left(w_{max}-w_{min}\right)\;\left[J/g_{ads}\right]$$

where:

- *H_ads_* [*J*/*g_ads_*] is the enthalpy of adsorption, which can be considered as the achievable heat storage density at a material level;
- Δ*H_ads_* [*J*/*g_water_*] is the differential enthalpy of adsorption referred to the adsorbed amount of water;
- *w_max_* and *w_min_* [*g_water_*/*g_ads_*] are the maximum and minimum adsorption amount of water over the adsorbent material, at the given working boundary conditions.

The value of the differential enthalpy of adsorption is generally calculated through the measurement of the equilibrium adsorption curves according to the well-known Clausius-Clapeyron equation [15]. Authors in [14] demonstrated that SAPO-34 and SWS generally show the highest heat storage capacities regardless of the boundary conditions. Nevertheless, classical zeolites whose integral heat of adsorption is very limited at 90 °C of regeneration temperature, become very attractive when higher temperatures (i.e., 160 °C) are available instead. Silica gels, as discussed previously, maintain quite a limited TES density, which makes this class of adsorbents less attractive for this application.

Finally, it must be emphasized that these calculations have been performed for unit mass. Usually, TES density is calculated on a volumetric basis, since the occupied volume can be an issue, for instance, in the domestic sector. Nevertheless, to calculate the volumetric adsorption storage is quite complicated, since the bulk adsorbent material density strongly depends on grain size and composition. For this reason, in order to compare different adsorbent materials, the gravimetric TES density is taken as a reference parameter. Main features of the introduced sorption material classes are shown in Table 1.

### 3.3. Adsorption TES Components

The development of closed adsorption TES components is mainly focused on the core component represented by the adsorber unit, realized by putting the adsorbent material in contact with an efficient heat exchanger. Indeed, since the adsorbent materials present a very low thermal conductivity, their inertia towards thermal cycles is quite high. This behavior is reflected in a limited dynamic performance which causes a reduced achievable specific power (both in terms of mass and volume), leading to bulky components. With regards to this, several activities have been carried out in the past to optimize the thermal conductivity of adsorbent materials by adding pieces of highly conductive materials [16]. More recently, research activity has been oriented mainly towards the optimization of the adsorber unit itself, to reduce the heat and mass transfer resistance between the heat exchanger and the adsorbent material. Thus far, three main technologies have been identified to realize effective adsorbers: Loose grains, binder-based coating, and in-situ crystallization coating techniques.

The less expensive and widely employed technique for adsorption TES is that which is based on loose grains embedded in the heat exchanger (HEX). In this case, the main goal is to find the best compromise, in terms of grain size, which allows for a good heat-transfer efficiency without significantly affecting the vapor diffusion through the adsorber.

The binder-based coating technique, is based on the reduction of contact resistance between the heat exchanger and the adsorbent material by distributing a thin, homogeneous layer over the wide heat transfer area of the HEX itself. In this way, heat transfer is enhanced, since the contact between the adsorbent and HEX is uniform and not punctuated, as in the loose grains configuration. Recently, potentialities of coatings on advanced HEX supports (graphite plates) have also been investigated [17]. Experimental results demonstrated that this approach could enhance the kinetic performance of the components, thus increasing the power of the developed units. Nevertheless, some parameters need to be carefully investigated in order to optimize the binder-based coating technique. Indeed, the adsorbent layer thickness needs to be carefully controlled, to avoid excessive mass-transfer resistance and to reach a high mechanical stability level. Furthermore, despite their good mechanical properties, organic binders can release small quantities of non-condensable gases, which can affect the performance of the adsorber itself.

The last option, currently under development, is the in-situ crystallization technique. This is mainly oriented towards zeolite and zeo-like materials, which are crystalline and can be directly synthesized over the metallic substrate of the HEX, leading to a perfect thermal contact, dramatically reducing the heat transfer resistance. This technique has already been applied to full-scale adsorbers and confirmed to be very promising from a dynamics point of view [18]. The main limitations, still under investigation, are related to the long duration and high energy consumption of the crystallization process, and to the low amount of adsorbent that can be deposited over the HEX, which can affect the achievable volumetric power.

### 3.4. Adsorption TES Systems

Adsorption TES systems are still in the early stages of development and are not yet completely commercialized. Nevertheless, some particular applications have already been put on the market because they fit certain needs perfectly. In this paragraph a brief collection of recently developed adsorption TES applications is reported.

**Figure 8:**
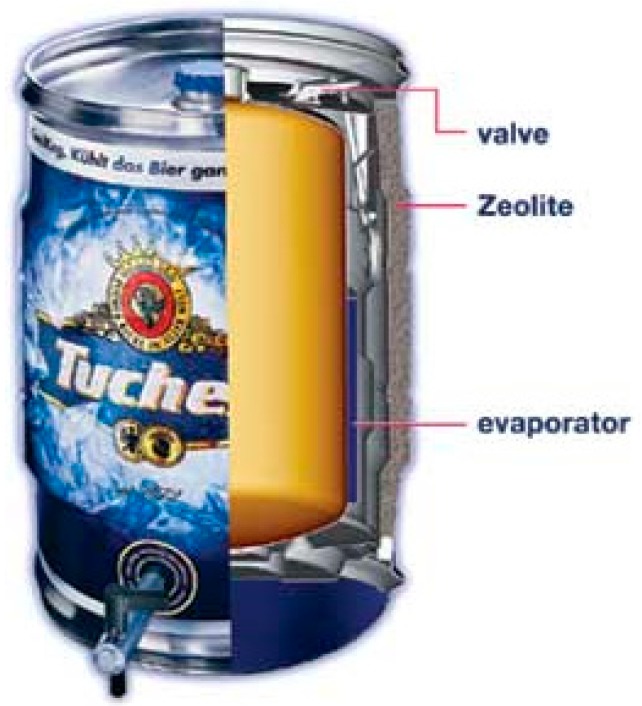
A small beer barrel with embedded adsorption TES for cooling. Courtesy of Cool-System (figure adapted from [19] with permission of Springer, 2007).

A classic example is the self-cooling portable beer barrel developed by ZeoTech company [19]. This system perfectly exploits the peculiarity of the adsorption TES. Indeed, it consists of a zeolite embedded inside the external shell of the barrel, kept separate from the evaporator (Figure 8). Once the beer needs to be cooled down, the connection between the anhydrous zeolite and the evaporator is opened through a manual valve, and the heat of evaporation is subtracted from the beer, which is cooled down to the desired temperature. When the barrel is empty, the saturated zeolite is regenerated in the oven, thus performing the charging phase. This process perfectly applies adsorption heat storage to small-scale apparatuses.

**Figure 9:**
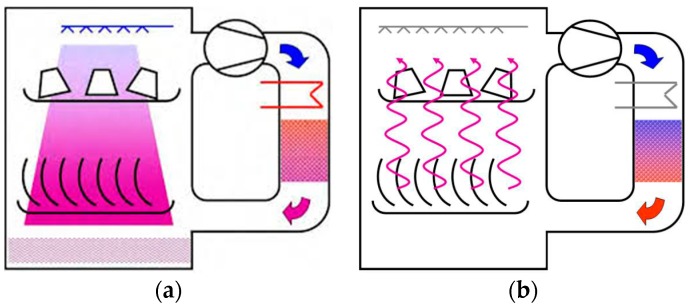
An adsorption based dishwasher. Washing phase (**a**); drying phase (**b**).

A different application of adsorption TES was recently developed and commercialized by Bosch in collaboration with ZAE Bayern, in which an open adsorption TES has been optimized to enhance the energetic performance of a dishwasher (Figure 9). The working principle can be found elsewhere [20]. Essentially, in this application, the thermal energy is reversibly stored in a zeolite cartridge, which is regenerated (charged) in the first phase of the washing process and discharged, with the release of a large amount of heat, during the drying phase. In this way, it becomes possible to reduce the amount of electrical energy required to perform an entire washing cycle, enhancing the energetic class of the appliance.

**Figure 10:**
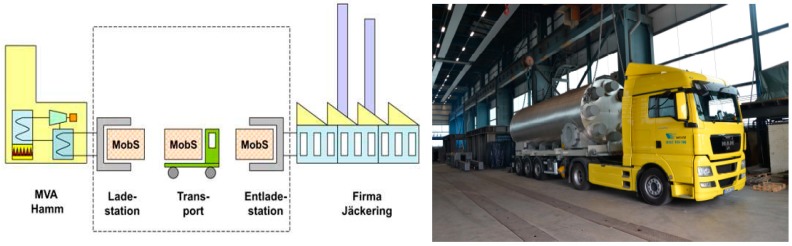
Mobile adsorption TES for industrial applications.

Another reported example of adsorption TES, is a large-scale system for industrial heat recovery, storage, and transportation, based on an open adsorption cycle. Figure 10 summarizes this concept, developed at ZAE Bayern laboratories [21]. It consists of recovering heat from an industrial site, by flowing hot air through a zeolite 13X bed. Once the adsorbent material is regenerated, the reactor full of dried zeolite (charged TES) is transported to the site, where it is discharged by flowing humid air through the zeolite bed, thus releasing heat to drive another industrial process. This system proved to be quite promising as the demand and the user side are closely related, as it is well known that industrial sites are one of the major sources of waste heat worldwide [22]. Therefore, to increase the share of this type of application, it is necessary to carefully analyze the boundary conditions, in order to make the process economically feasible.

**Figure 11:**
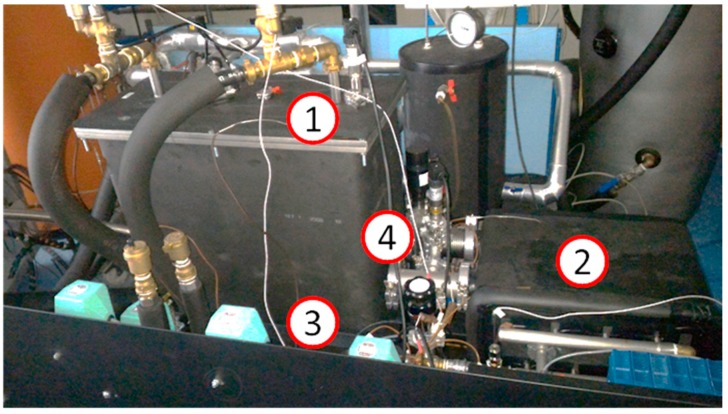
The TES prototype system realized at CNR-ITAE. 1: Adsorber, 2: Phase changer, 3: Hydraulics. 4: Vacuum valve.

The final example is a prototype of a compact TES for mobile applications [23]. The sorption TES has been realized and tested at CNR-ITAE. It consists of two vacuum chambers, for the adsorber and the phase changer (Figure 11). Certain flanges allow connection to the sensors for monitoring of the most relevant parameters (pressure, temperature) and the other components of the device. The exchanger used for the adsorber is a flat-tube and fins-type with an exchange area of 1.75 m^2^. The exchanger has been filled with 4.3 kg of AQSOA™-Z02 grains, in the range 1–2 mm. The material has then been contained by means of a metallic net. The connection between the adsorber and the phase changer is realized through an electrically actuated pneumatic valve.

The phase changer consists of a welded chamber, containing four high efficiency fin-and-tube heat exchangers with copper fins and stainless steel tubes connected in parallel through an external tubular steel manifold. Each one has an exchange surface of 1.33 m^2^. Vacuum flanges allow connections to the adsorber and to some sensors.

The test set-up is completed by a hydraulic circuit realized with copper Φ12 mm tubes, thermally insulated by a polyurethane foam with a thickness of 1.5 cm. The hydraulic circuits include four 3-way valves.

The results obtained with this system showed good performance and efficiency, consistent with those recently measured by others and theoretically predicted [24,25]. In particular, storage capacities up to 263 Wh/kg of adsorbent were obtained, corresponding to a storage capacity 40% higher than that of water under the same boundary conditions. A peculiarity of the investigated system is the possibility to use low-temperature waste heat (T < 100 °C) both for heat and cold storage purposes: the measurements carried out highlighted that even with a heat source temperature at 85 °C, temperatures of 5–10 °C can be efficiently produced.

## 4. Conclusions and Future Perspectives

The present paper has summarized some of the main features of the adsorption TES, analyzing its state of development from different points of view, from the material up to the system applications. Research activity is still ongoing, in an attempt to solve the main issues related to this technology. Particularly, the following ways seem to be quite promising in order to reach commercial diffusion during next years:

- At a material level, the main challenge is to reduce the costs of available materials, in order to make the adsorption TES systems more competitive. In this context, a lot of efforts are put into the employment of less expensive raw materials for zeo-like adsorbents and to reduce the hydrophilicity of classical zeolites, to keep down the required regeneration temperature. Furthermore, MOFs are continuously under development, thanks to their promising features.
- At a component level, the main task is the realization of efficient adsorbers, based either on adsorbent coating or on a loose grains technique, which can allow the size of the storage systems to be limited, enhancing the kinetic performance. Furthermore, particular attention is also placed on the reliability of these components, in terms of corrosion and hydro-thermal stability issues.
- At a system level, small-scale adsorption TES units are under development for domestic applications. Indeed, if properly coupled to the distribution system, they can not only store thermal energy, but also provide a heat-pumping effect during the winter season and cooling energy during the summer season, thus making this a component for fully-integrated heat and cold storage, throughout the year.

## Figures and Tables

**Table 1:** Main properties of sorbent material classes.

	Silica Gels	Zeolites	ALPO_S_/SAPO_S_	Composites	MOFs	Activated Carbons
**Adsorption heat (kJ/kg)**	160 ÷ 180 *	50 ÷ 300*	250 ÷ 300 *	50 ÷ 250*	20 ÷ 200 **	45 ÷ 900 ***
**Typical desorption temperatures [°C]**	50 ÷ 80	70 ÷ 350	60 ÷ 90	60 ÷ 90	60 ÷ 150	80 ÷ 200
**Density (kg/m^3^)**	650 ÷ 700	650 ÷ 900	800 ÷ 900	300 ÷ 600	1000 ÷ 2000	700 ÷ 750
**Specific heat (kJ/kgK)**	0.8 ÷ 0.9	0.85 ÷ 0.95	0.85 ÷ 0.95	0.95 ÷ 1.05	0.8 ÷ 1.2	0.8 ÷ 1.5
**Thermal conductivity (W/mK)**	0.15 ÷ 0.20	0.15 ÷ 0.25	0.15 ÷ 0.25	0.15 ÷ 0.30	0.10 ÷ 015	0.15 ÷ 0.75
**Possible refrigerants**	water	water	water	water, methanol, ethanol	water, methanol, ethanol	methanol, ethanol, ammonia
**Amount of uptake exchanged in a typical cyle [g/g]**	0.03 ÷ 0.10	up to 0.2	up to 0.25	up to 0.8	0.16 ÷ 0.40	015 ÷ 0.60

* the heat of adsorption is calculated for a cycle with T_des_ = 100 °C, T_cond_ = 30 °C, T_ads_ = 50 °C, T_ev_ = 10 °C, with water as sorbate; ** the heat of adsorption is calcualted from isotherms at 298 K, 303 K and 333 K, with water as sorbate; *** the range of heat of adsorption is calculated with methanol and ammonia as sorbates.
